# Anaesthesiologists Under Strain: Burnout and Its Associated Factors in a Conflict-affected Context, a Cross-sectional Study

**DOI:** 10.4274/TJAR.2026.262471

**Published:** 2026-10-30

**Authors:** Vanda Yazbeck Karam, Zeina Akiki, Wael Salame, Georges Assaf, Carole Serhal, Samantha Succar, Rony Al Nawwar, Sandra Elias, Jessy Abou Nader, Charbel Gerges, Micheline Boukhalil, Hanane Barakat

**Affiliations:** 1 Gilbert and Rose-Marie Chagoury Faculty of Medicine, Lebanese American University, Byblos, Lebanon; 2 Gilbert and Rose-Marie Chagoury Faculty of Medicine, Department of Anaesthesiology, Lebanese American University, Byblos, Lebanon; 3 INSPECT-LB (Institut National de Santé Publique, d'Épidémiologie Clinique et de Toxicologie-Liban), Beirut, Lebanon

**Keywords:** Anaesthesiologists, client-related burnout, conflict-affected region, personal burnout, work-related burnout

## Abstract

**Objective:**

Anaesthesiologists are particularly vulnerable to burnout due to extended working hours, high-pressure situations, limited patient interactions, multiple responsibilities, and inherent tensions in relationships with surgeons and administrators. The study aimed to explore burnout levels and the factors associated with them among anaesthesiologists in a conflict-affected setting.

**Methods:**

A cross-sectional study was carried out between May 2024 and August 2025 using a voluntary, anonymous, and electronic survey. The Copenhagen Burnout Inventory, demographics, and practice environment questions were included. Statistical analyses were conducted to identify factors associated with personal, work-related, and client-related burnout. A significance threshold of 0.05 was applied.

**Results:**

Among the 82 anaesthesiologists, 57% experienced personal burnout, 51% experienced work-related burnout, and 28% experienced client-related burnout. Positive and significant associations were observed between working longer hours and personal burnout (adjusted β [95% confidence interval (CI)]=15.6 (0.88; 30.3); *P*=0.04) and work-related burnout [adjusted ß (95% CI)=17.7 (3.68; 31.7); *P*=0.01], as well as between covering five or more night shifts per month and client-related burnout [adjusted ß (95% CI)=15.3 (3.86; 26.7); *P*=0.009].

**Conclusion:**

The findings reveal concerning levels of burnout among anaesthesiologists in conflict-affected regions, which is particularly important given the ongoing regional conflict and limited conflict-specific data, and highlight the potential role of modifiable predictors and associated factors. Targeted interventions are essential to support anaesthesiologists’ well-being and ensure the effective functioning of healthcare institutions in such settings. Future studies with larger, randomly selected samples are warranted to further explore this issue.

**Main Points**

• High levels of burnout were observed among anaesthesiologists, with personal and work-related burnout affecting more than half of participants, and client-related burnout affecting over one-quarter of participants.

• Workload-related factors were independently associated with burnout, with longer working hours associated with increased personal and work-related burnout, and frequent night shifts (≥5 per month) with higher client-related burnout.

• The findings underscore the cumulative psychological burden of practicing in a conflict-affected setting, where prolonged instability may exacerbate occupational stress.

• Interventions targeting modifiable work conditions are warranted to mitigate burnout and support anaesthesiologists’ well-being; further research using larger, randomized samples is also warranted.

## Introduction

The demanding requirements and excessive levels of stress among healthcare professionals, especially physicians, often place a strain on their mental and physical wellbeing, making them more vulnerable to burnout.^1^ Anaesthesiologists are particularly susceptible to burnout due to prolonged working hours, high-pressure environments, and frequent exposure to critical situations.^2, 3, 4^ Additionally, multiple responsibilities, including clinical, academic, research, and administrative duties, coupled with limited patient interaction and insufficient compensation, can further exacerbate burnout.^5, 6^ The inherent tension in relationships with surgeons and interventionalists can compound the psychological strain experienced by anaesthesiologists.^5, 7^

Burnout is a condition of persistent physical and psychological fatigue resulting from chronic stress, often affecting personal, work, and client-related areas.^8^ Burnout can compromise patient care by causing delayed decisions, impaired judgment, poor communication, and inadequate record-keeping, all of which increase the risk of adverse events.^9^ Previous research indicated that 34% of medical school faculty suffered from emotional exhaustion, 66.3% from depersonalization, and 92.4% from reduced personal accomplishment, while 30.5% exhibited high burnout in all dimensions.^10^ A systematic review conducted among anaesthesiologists found a prevalence ranging from 10 to 41% of high-risk of burnout, and up to 59% of at least moderate risk.^2^ While the exact prevalence of burnout among anaesthesiologists remains hard to determine in low- and middle-income countries (LMICs) due to methodological limitations, studies suggest that a significant proportion may be affected.^11, 12, 13^ Factors positively associated with burnout were long working hours, working as a younger practice, and having children; while no associations were observed with gender or marital status.^2^ Moreover, anaesthesiologists in LMICs face additional challenges that contribute to burnout. Heavy workloads, resource shortages, and low income can take a toll on LMICs anaesthesiologists‘ physical and mental well-being, in addition to their personal life outcomes.^13, 14, 15^

Lebanon has experienced unprecedented crises since 2019, encompassing the coronavirus disease-19 pandemic with its mental health repercussions, the economic meltdown including sharp currency devaluation and banking sector deterioration, and the aftermath of the 2020 Beirut Port explosion. These crises have resulted in soaring inflation, scarcities of essential goods, and an exodus of skilled labor, including healthcare professionals. Political corruption and historical conflicts have compounded the strain on the economy, deepening unemployment and poverty amid persistent social unrest.^16, 17, 18^ Moreover, in late 2024, bombings in Beirut and other regions further heightened stress among Lebanese healthcare workers.^19^ In 2025, burnout levels among Lebanese residents and fellows were assessed using the Maslach Burnout Inventory (MBI), revealing that 58% of participants experienced high levels of emotional exhaustion, 28% demonstrated high depersonalization, and 50% noted low personal accomplishment.^20^ Among anaesthesiology residents, 71% reported personal burnout, 68% reported work-related burnout, and 36% experienced client-related burnout.^19^ Additionally, earlier data from 2020 showed that 19% of surveyed physicians experienced high burnout levels.^21^ While these studies confirm that burnout is prevalent among healthcare professionals in Lebanon, they predominantly include participants from mixed specialties or focus on trainees, and do not capture the specific occupational exposures and stressors unique to attending anaesthesiologists. Furthermore, existing literature has largely examined burnout in stable or single-crisis settings, without accounting for the compounded and prolonged impact of overlapping economic collapse, workforce migration, and active conflict.

Anaesthesiologists represent a particularly relevant group to study in this context, as their role extends beyond routine perioperative care to include critical care management, emergency response, and involvement in mass casualty incidents, responsibilities that are amplified in conflict-affected and resource-constrained environments.^22^ Despite this, there remains a lack of specialty-specific evidence assessing burnout and its determinants among anaesthesiologists practicing under such compounded stressors. Therefore, this study addresses a critical gap by providing a specialty-specific evaluation of burnout among anaesthesiologists practicing within a context characterized by overlapping crises, workforce depletion, and heightened clinical demands. By examining both individual and system-level determinants, this work offers a more comprehensive perspective on burnout compared to prior studies.

Thus, this study aimed to investigate the level of burnout. Specifically, it to (1) assess burnout using a validated multidimensional tool, 2) identify occupation- and context-specific factors associated with burnout, including workload and work-environment variables and 3) generate context-informed recommendations that may be transferable to other conflict-affected or resource-limited settings.

## Methods

### Study Design

A cross-sectional study was conducted among anaesthesiologists practicing in Lebanon between May 2024 and August 2025. Anaesthesiology was selected because of its high exposure to occupational stressors, critical care workload, and perioperative decision-making demands, which make it a high-risk specialty for burnout. In the Lebanese context, this specialty is particularly affected by post-crisis health-system strain and workforce migration, which have increased workload pressures and staffing shortages, making this population particularly relevant for burnout assessment.

### Sample Size Calculation

In 2024, the Lebanese Society of Anaesthesiologists database included approximately 400 anaesthesiologists. In Lebanon, a prior study on physician burnout indicated that 90.1% met the established criteria.^21^ Based on calculations using Epi Info software, a minimum of 78 participants was required to ensure a 90% confidence interval, and a 5% margin of error for the expected burnout prevalence estimate. A total of 82 anaesthesiologists participated in the study; the final sample represents approximately 20.5% of the known anaesthesiology workforce in Lebanon. However, because recruitment was conducted using snowball sampling, the number of individuals who received or viewed the survey invitation could not be determined, and a formal response rate could not be calculated.

### Data Collection, Patient Consent and Ethical Approval

A web-based questionnaire in English was developed using Google Forms and pilot-tested with five anaesthesiologists. The final version was distributed to participants through a snowball sampling approach. At the beginning of the survey, the study’s objectives and scope were clearly explained, and participants were informed that their participation was voluntary, anonymous, and confidential. The informed consent procedure required participants to click the survey link, which directed them to a consent page. To access the survey, participants had to click “yes” to indicate their consent, while selecting “no” terminated the survey (Patient Consent Form). The study was conducted online and was granted exemption by the Lebanese American University Institutional Review Board (IRB tracking number: LAU.SOM.VA2.21/May/2024; date: 21 May 2024; IRB Notice of Exemption). This online approach was particularly appropriate given the post-crisis context in Lebanon, where geographic dispersion of physicians, time constraints due to increased clinical burden, and migration-related workforce instability limited the feasibility of in-person data collection.

### Measurement Tools

The questionnaire was divided into 2 sections: 1) demographic and practice-related questions and personal and occupational risk factors for burnout; and 2) burnout, determined using the Copenhagen Burnout Inventory (CBI).

### Description of the CBI

The CBI is a validated instrument comprising three parts: personal burnout, work-related burnout, and client-related burnout. Personal burnout items assess general fatigue and exhaustion, while work-related burnout items assess perceived burnout related to their job among individuals in paid employment. Client-related burnout items measure burnout associated with working with clients (referred to as patients in this study). The CBI consists of 19 items, with 6, 7, and 6 questions addressing personal, work-related, and client-related burnout, respectively. Responses are recorded on a five-point Likert scale and scored from 0 (never, almost never, or to a very low degree) to 100 (always or to a very high degree). Although the CBI authors did not specify cut-off scores, several studies have categorized burnout using a threshold of <50 versus ≥50^21, 23^ and ≥75 reflecting high burnout levels.^24, 25, 26^

### Statistical Analysis

The CBI subscales were computed, and descriptive statistics were used to summarize participants’ characteristics and subscale scores. Continuous variables were reported as mean ± standard deviation (SD) or median with interquartile range, as appropriate, while categorical variables were expressed as percentages. Internal consistency of the CBI subscales was assessed using Cronbach’s alpha, and correlations among subscales were examined. The normality of quantitative variables was examined using the Shapiro-Wilk test and visually assessed using histograms.

For bivariate analyses, Student’s t-test was used to compare means between two groups, and ANOVA was used to compare means across three or more groups after verifying homogeneity of variances with Levene’s test. For subgroups with fewer than 30 participants or with non-normally distributed continuous variables, non-parametric tests such as the Kruskal-Wallis test were employed. Pearson correlation coefficients were used to test associations between continuous variables when assumptions of normality and linearity were satisfied.

Multivariable linear regression analyses were conducted to assess associations between the CBI subscales and potential confounders after verifying the assumptions of normality (of dependent variables or residuals), homoscedasticity, linearity, and the absence of multicollinearity. Three separate regression models were fitted, with personal, work-related, and client-related burnout as dependent variables. Variables with a *P* < 0.2 in the bivariate analysis were included as covariates (e.g., weekly working hours). Regression coefficients (β) and 95% confidence intervals (CIs) were reported to quantify these associations. Statistical significance was set at α=0.05. All analyses were performed using IBM SPSS Statistics version 29 (IBM Corp., Armonk, NY, USA).

## Results

### Participants’ Characteristics

This study included 82 anaesthesiologists, whose mean age was 48 years. Around half were female, and 81% were married or in a relationship. Moreover, 45% were exercising, and 67% were non-smokers. Around half reported experiencing chronic diseases, and 22% reported mental health problems (Table 1).

### The Practice Environment

The majority (65%) were affiliated with a university hospital, and 51% had over 16 years of experience. In addition, 59% worked over 40 hours per week, and 61% covered 5 or more night shifts each month (Table 1). Additional variables related to the perceived work environment, including staffing shortages and support systems, are presented in Supplementary Figure 1 (Participants’ concerns).

### Burnout Across the CBI Subscales

Cronbach’s alpha for the CBI subscales was 0.90 for personal burnout, 0.90 for work-related burnout, and 0.67 for client-related burnout, indicating acceptable to excellent internal consistency. In addition, the proportion of burnout was found to be 57.3% (95% CI=45.9%-68.2%), 51.2% (95% CI=39.9%-62.4%) and 28% (95% CI=18.7%-39.1%) for personal (mean ± SD=51.6±20.1), work-related (mean ± SD=49.7±21.5) and client-related burnout (mean ± SD=35.5±16.3) respectively. High burnout levels (score ≥75) were observed for personal [17.1% (95% CI=8.5%-24.4%)], and work-related burnout [14.6% (7.3%-22%)]. A strong positive correlation was observed between personal and work-related burnout; moderate positive correlations were observed between personal and client-related burnout and between work-related and client-related burnout (Table 2).

### Factors Associated with CBI Subscales

### Personal Burnout

The bivariate analysis showed that anaesthesiologists who experienced personal burnout were younger and less physically active. Moreover, they tended to report more mental health problems, to have fewer years of experience, and to work longer hours, particularly those working 50 hours per week or more. They also covered more night shifts (specifically more than 5 per month) and took fewer vacation days (Table 3). In the multivariable analysis, and after adjusting for covariates, working ≥50 hours in a week was significantly associated with a 15.6-point higher personal burnout compared to working fewer than or equal to 40 hours (95% CI=0.88, 30.3; *P*=0.04) (Table 4).

### Work-related Burnout

Anaesthesiologists who experienced work-related burnout were younger, more likely to be smokers and to report mental health problems. Moreover, they were mostly affiliated with community hospitals, tended to have fewer years of experience, and worked longer hours; this was especially true for those working 50 hours per week or more. They also covered more night shifts, specifically 5 per month or more, and took fewer vacation days (Table 3). In the multivariable analysis, working ≥50 hours in a week was significantly associated with a 17.7-point higher work-related burnout compared to working fewer than or equal to 40 hours (95% CI=3.68, 31.7; *P*=0.01) (Table 4).

### Client-related Burnout

Anaesthesiologists experiencing client-related burnout were more likely to be younger, have longer working hours, and cover more night shifts, specifically if they worked 5 or more night shifts per month (Table 3). In the multivariable analysis, covering 5 or more night shifts per month was significantly associated with a 15.3-point higher client-related burnout score compared with not covering night shifts (95% CI=3.86, 26.7;* P*=0.009) (Table 4). These findings inform the practical considerations outlined in Table 5.

## Discussion

This study, among the first to examine burnout among attending anaesthesiologists in Lebanon, a conflict-affected region, within the context of ongoing economic and political crises and regional conflict, found high burnout levels, with personal and work-related burnout affecting over half of participants and client-related burnout affecting over one quarter. Longer working hours and frequent night shifts (≥5/month) were significantly associated with higher burnout across these domains.

Burnout among anaesthesiologists appears more widespread than reported in other countries. A 2021 study conducted in the United States and using the MBI, reported that 13.8% met the criteria for burnout, while 59.2% were at high-risk for burnout.^27^ Another 2023 study conducted among French anaesthesiologists and using CBI reported lower proportions with 24% experiencing burnout.^5^ A 2017 systematic review indicated that 10-41% of anaesthesiologists are at high-risk of burnout, with up to 59% experiencing at least moderate risk. In addition, burnout among anaesthesiologists has been reported as prevalent in LMICs, with proportions reaching 51.3% among anaesthesiologists in Zambia and 62.2% for emotional exhaustion among those in Egypt.^13, 15^ While previous studies report varying rates of burnout among anaesthesiologists, influenced by study design, location, and collective stressors,^28^ our findings suggest a comparatively higher burden of burnout within a conflict-affected and economically strained setting, thereby extending existing evidence by contextualizing burnout within compounded crises rather than isolated occupational stressors.

In addition, a 2020 study assessing burnout among physicians in Lebanon and using CBI reported that 90% of participants were experiencing burnout, with high levels observed in 19.1% of them.^21^ Unlike previous work, our study focuses specifically on anaesthesiologists and provides a specialty-specific perspective with more homogeneous exposures, allowing a clearer identification of profession-related drivers of burnout. However, there is consistency in the ranking of dimensions: personal burnout ranked first in both studies. This consistency reinforces the relevance of personal-level stress even across heterogeneous and specialty-specific samples. As the conflict persists, burnout among anaesthesiologists may worsen, threatening both physician well-being and the quality, safety, and sustainability of healthcare in fragile settings.

This study found that working more than 50 hours per week was positively associated with work-related and personal burnout, consistent with previous reports. Actually, long working hours were significantly associated with a poor mental health, chronic stress and a higher incidence of burnout.^29, 30, 31^ Long working hours may increase job demands, such as night calls and irregular schedules, leading to physical and mental fatigue and leaving fewer opportunities for rest, particularly when these demands are imposed and accompanied by a reduced sense of autonomy. Those extended working hours might impair the body’s ability to restore energy and often require sustained attention, cause decision-making fatigue, and lead to errors, further exacerbating burnout. Besides, they may restrict time for family, social activities, and recovery, leading to dissatisfaction.^32, 33^ Importantly, this study raises the possibility that the observed extended working hours are partly linked to the ongoing emigration of physicians from Lebanon, a structural factor that is driven by the economic crisis and is rarely quantified in burnout research. The outflow of trained professionals has increased workload among those remaining, requiring longer shifts and expanded responsibilities.^34^ This highlights how workforce depletion may act as a system-level driver of burnout, distinguishing our findings from studies conducted in more stable healthcare systems.

On the other hand, long working hours were not associated with client-related burnout. This finding provides a nuanced, specialty-specific insight, as patient interaction might not be the primary driver of burnout among anaesthesiologists. Unlike other specialties that involve prolonged direct patient interactions, such as oncology or psychiatry, anaesthesiologists’ patient contact is intense but limited. In fact, stress is more likely driven by technical demands, long operating room hours, and on-call duties rather than emotional strain from patient interactions.^2, 3^

In addition, taking more than 5 night shifts per month was associated with client-related burnout. Previous reports showed that the odds of burnout increased by 3 to 9 percent for every additional night on call per week.^1^ In our context, this association might be further compounded by workforce shortages due to physician migration, which increases the frequency of night duties among remaining staff.^35^ Night shifts disrupt the circadian rhythms, leading to sleep deprivation, and eventually compromising attention and emotional regulation.^36, 37^ Our findings suggest that under such strained conditions, even brief patient interactions may become more psychologically demanding, particularly when staffing is reduced during night hours.^38^

Additionally, younger age and fewer years of professional experience were significantly associated with higher levels of personal and work-related burnout. However, these associations did not reach statistical significance in the multivariable models. Previous studies reported that younger anaesthesiologists, particularly those early in their careers, are more vulnerable to burnout.^2, 39^ Anaesthesiologists with fewer years of experience may have limited autonomy or may feel uncertain about independently managing difficult situations, such as airway difficulties or cardiac arrest. In conflict-affected countries, this challenge is amplified, as early-career anaesthesiologists may be required to assume responsibilities in crisis or resource-limited environments without sufficient preparation or mentorship, an aspect that remains underexplored in the literature.^4^ This may also reflect a “surviving effect”, as described by Downey et al.,^40^ where those who remain in the profession develop coping strategies over time. Thus, those remaining may appear less impacted by stress compared to younger or newer anaesthesiologists.^2, 40^ This study included a relatively small number of anaesthesiologists with fewer years of experience, which may explain why working hours emerged as a stronger predictor of burnout, whereas age and years of experience were not significant in the regression model.

No significant association was found between gender and burnout, consistent with prior systematic reviews reporting mixed findings.^2^ This supports the interpretation that structural and workplace-related factors may outweigh individual characteristics in determining burnout in high-stress and crisis.^41^ In the Lebanese context, socio-cultural dynamics, economic instability, and the stigma surrounding mental health likely affect male and female anaesthesiologists similarly. Furthermore, strong female leadership and visibility within Lebanese anaesthesiology may foster a supportive professional environment, potentially reducing gender-related disparities in burnout.^16^ Collectively, these observations indicate that while gender may play a role in burnout at a global level, in conflict-affected settings, working conditions may be more decisive in shaping burnout among anaesthesiologists.

Moreover, this study found no association between physical activity among anaesthesiologists and burnout levels in the regression analysis. This finding contrasts with previous studies reporting that physically active healthcare workers are at lower risk of burnout.^42^ The mechanisms of this association may be explained by the release of endorphins, improved sleep quality, and enhanced overall physical health, which collectively promote mental well-being and reduce burnout risk.^42^ Our findings indicate that in high-burden environments, occupational stressors such as workload intensity and staffing shortages may overshadow individual-level protective behaviors. We believe that, among anaesthesiologists, working hours, night shifts, and workload intensity are the main drivers of burnout. These occupational demands may outweigh the potential protective effects of physical activity, making the association statistically insignificant. Moreover, physical activity was self-reported; data on the type of activity were not collected, which may affect its observed association with burnout.

### Practical Implications

By integrating both occupational and structural determinants of burnout, this study provides a more comprehensive framework for intervention compared to prior research. The authors propose multi-level strategies targeting policy-makers, institutions, and individuals to mitigate burnout.^43^

### Study Limitations

This study is among the first to assess burnout levels in attending anaesthesiologists in the context of Lebanon’s ongoing economic and political crises and regional conflict. Its primary strength is its context-specific contribution, which captures the combined effects of workforce migration, systemic instability, and high clinical demand, factors that are rarely examined together in the existing literature. These findings may have broader implications for anaesthesiologists working in other conflict-affected or resource-constrained settings, where overlapping crises amplify professional stress and burnout. Importantly, the study did not include conflict-specific variables (e.g., displacement or direct exposure to mass casualty events), because all participants were indirectly exposed to a shared conflict-affected healthcare environment, which limited the ability to examine heterogeneity in individual conflict exposure. The non-random snowball sampling method and relatively small sample size may have introduced selection bias and may not fully represent the broader population of anaesthesiologists. Participants who were more professionally connected, more engaged in professional networks, or more motivated to participate because of personal experiences with burnout may have been overrepresented. Consequently, the burnout estimates should be interpreted with caution, as levels may have been overestimated or underestimated, and overestimation is plausible if individuals experiencing greater burnout were more likely to respond. Although the study achieved the minimum sample size required under the predefined assumptions and a 90% confidence level, a larger sample would have been required had the more conventional 95% confidence level been used. Therefore, the relatively small sample size may have reduced the precision of some estimates and limited the statistical power to detect smaller associations, warranting cautious interpretation of the findings. Therefore, the findings may not be fully generalizable to all anaesthesiologists practicing in Lebanon. Moreover, because snowball sampling was used, the total number of anaesthesiologists who received the survey is unknown, and a true response rate cannot be calculated. Although 82 participants represented about 20.5% of the estimated workforce, this does not constitute a response rate. Selection bias may exist, as participants could differ from non-participants, limiting generalizability. The cross-sectional design precludes the establishment of causal relationships, and the reliance on self-reported data may have introduced information bias. This potential bias was likely mitigated by the use of validated instruments, such as the CBI. Although relevant covariates were included in the analyses, the possibility of residual confounding from unmeasured variables cannot be excluded. Although analyses were conducted on the predefined CBI subscales and were guided by a priori hypotheses, the possibility of type I error due to multiple comparisons cannot be excluded. Therefore, findings, particularly those with borderline statistical significance, should be interpreted with caution.

## Conclusion

This study highlights concerning levels of burnout among anaesthesiologists, particularly in the context of an ongoing and intensifying regional conflict. However, it adds value by situating these findings within the current Lebanese context of compounded systemic strain, including prolonged economic instability, workforce depletion, and escalating regional conflict. In this setting, anaesthesiologists work under increasingly constrained conditions, which may exacerbate established risk factors for burnout. Nevertheless, the key predictors and associated factors identified appear to be modifiable. Implementing targeted interventions to reduce occupational stress, such as enforcing a maximum 48-hour workweek, limiting consecutive night shifts, promoting access to mental health support, and encouraging self-care practices, may be essential for safeguarding anaesthesiologists’ well-being and maintaining the effective functioning of healthcare institutions in fragile settings. Future research should incorporate randomly selected samples and longitudinal designs to monitor burnout trends over time in crisis-affected health systems. Additionally, interventional studies are warranted to evaluate the effectiveness of institutional support measures, wellness programs, and preparedness training initiatives.

## Ethics

**Ethics Committee Approval:** The study was conducted online and was granted exemption by the Lebanese American University Institutional Review Board (IRB tracking number: LAU.SOM.VA2.21/May/2024; date: 21 May 2024).

**Informed Consent:** Participants were informed that their participation in the study was voluntary, and they were assured that their responses would remain anonymous and confidential. Informed consent was obtained from all of the participants before completing the questionnaire.

## Supplementary Materials

Supplementary Figure https://d2v96fxpocvxx.cloudfront.net/688d2d00-d207-464d-89b6-73f393f4f50c/content-images/7b4f0de7-0f7c-4c7b-ab4a-a65df7436d68.pdf

## Figures and Tables

**Table 1. Participants’ Socio-Demographic Characteristics and Working Environment (n = 82):** 

-		**n (%)**
**Gender**	Female	43 (52%)
	Male	39 (48%)
**Age;** **mean** **±** **SD**	-	48.2±11.8
**Minimum-maximum**	-	28-83
**Relationship** **status**	Single/not in a relationship	16 (19%)
	Married or in a relationship	66 (81%)
**Smoking** **status**	Non-smokers	55 (67%)
	Smokers	20 (24%)
	Ex-smokers	7 (9%)
**Physical** **activity**	No or occasionally	45 (55%)
	1 to 2 times per week	29 (35%)
	3 times or more per week	8 (10%)
**Chronic** **diseases** ^#^	Yes %	42 (51%)
**Mental** **health** **problems** ^#^	Yes %	18 (22%)
**Primary** **practice** **environment**	University hospital/academic	53 (65%)
	Private practice	24 (29%)
	Community hospital	5 (6%)
**Years** experience^*^	0 to 5	15 (18%)
	6 to 15	25 (31%)
	16 or more	42 (51%)
**Working** **hours** **per** **week**	≤40	10 (12%)
	41 to 50	24 (29%)
	>50	48 (59%)
**Night** **shifts** **per** **month**	0	11 (13%)
	1 to 4	21 (26%)
	5 or more	50 (61%)
**Number** **of** **days’** **vacation** **per** **year**	≤14	16 (20%)
	15 to 21	24 (29%)
	22 to 30	42 (51%)

^#^As diagnosed by a physician; ^*^After last year anaesthesia training (including fellowship); SD, standard deviation; all results are presented as n (%) unless otherwise specified

**Table 2. CBI Burnout Dimensions or Subscales:** 

-	**Personal**	**Work-related**	**Client-related**
**Mean** **±** **SD**	51.6±20.1	49.7±21.5	35.5±16.3
**Minimum-maximum**	0-100	0-100	0-71
**Correlation** **among** **CBI** **burnout** **subscales**			
-	**Personal**	**Work-related**	**Client-related**
**Personal** **burnout**	-	r=0.85 ***P*** **<** **0.001**	r=0.47 ***P*** **<** **0.001**
**Work-related** **burnout**	-	-	r=0.49 ***P*** **<** **0.001**

Values in bold indicate statistical significance at *P < *0.05* r*, pearson correlation coefficient; *P*, *P *value

**Table 3. Bivariate Associations with CBI Burnout Subscales:** 

-		**Personal **		**Work-related**		**Client-related**	
		**(Mean ± SD) or r**	***P***	**(Mean ± SD) or r**	***P***	**(Mean ± SD) or r**	***P***
**Gender**	Female	50.1±21.4	0.5	47.4±21.5	0.3	35.6±16	0.9
	Male	53.2±18.6	-	52.3±21.4	-	35.4±16.8	-
**Age**	-	-0.43	**<0.001**	-0.47	**<0.001**	-0.26	**0.02**
**Relationship** **status**	Not in a relationship	52.1±17.5	0.9	48±20.7	0.7	33.3±15.1	0.6
	Married/In a relationship	51.5±20.8	-	51.2±21.8	-	36±16.6	-
**Smoking** **status**	Non-smokers	48.3±19.3	0.054	46.4±19.9	**0.01**	33.7±14.8	0.2
	Smokers	60.8±20.1	-	61.6±21.6	-	42.1±20.5	-
	Ex-smokers	51.2±20.4	-	42.3±23	-	30.9±8.28	-
**Physical** **activity**	No or occasionally	54.5±20.2	**0.04**	53.6±22.2	0.1	37.4±16.9	0.2
	1 to 2 times per week	51.4±17.6	-	47.2±16.9	-	35.3±15.3	-
	3 times or more per week	35.4±22.2	-	37.5±28.5	-	25.5±14	-
**Chronic** **diseases**	Yes	52.4±20.8	0.7	50±22.5	0.9	35.7±17.7	0.9
	No	50.7±19.5	-	49.5±20.6	-	35.3±14.9	-
**MH** **problems**	Yes	61.1±18.7	**0.02**	64.3±18.2	**<0.001**	37.7±16.4	0.5
	No	48.9±19.7	-	45.6±20.6	-	34.9±16.3	-
**Main** **practice** **type**	University hospital	49.6±17.4	0.08	49.2±19.8	**0.047**	34.8±17.3	0.2
	Private Community hospital	53.3±25.7 64.2±11.6	-	48.2±26.1 62.9±9.7	-	34.5±13.4 47.5±16	-
**Years** **Experience^#^**	0 to 5	66.1±12.7	**<0.001**	64.8±16.4	**<0.001**	39.2±14.2	0.2
	6 to 15	55±22.9	-	55±22	-	38.5±17.4	-
	16 or more	44.3±17.2	-	41.2±19	-	32.4±16	-
**Working** **hours** **per** **week**	≤40	36.3±16.2	**<0.001**	32.5±16.9	**<0.001**	27.1±11.2	**0.007**
	41 to 50	41.8±17.9	-	38.1±19	-	29.9±13.3	-
	>50	59.6±17.9	-	59.2±18.4	-	40.1±17.1	-
**Night** **shifts** **per** **month**	zero	37.5±18	**<0.001**	30.8±18.5	**<0.001**	22.3±12.7	**<0.001**
	1 to 4	41.1±17.9	-	36.7±19.9	-	27.8±15.6	-
	5 or more	59.1±16.9	-	59.4±16.8	-	41.7±14.2	-
**Days** **vacation** **per** **year**	≤14	63± 21.8	**0.009**	60.7±18.8	**0.009**	38±13	0.7
	15 to 21	54.2±19	-	54±22.7	-	36.3±16.1	-
	22 to 30	45.7±18.1	-	43.1±19.7	-	34.1±17.6	-

^#^After last year anaesthesia training (including fellowship), values in bold indicate statistical significance at *P* < 0.05; SD, standard deviation; *P*, *P* value; CBI, Copenhagen Burnout Inventory; MH, mental health

**Table 4. Multivariable Models for Each CBI Burnout Subscale:** 

**1^st^ regression. Personal burnout**		**Adjusted β (95% CI)**	***P***
**Age**	-	-0.13 (-0.84; 0.59)	0.7
**Smoking** **status**	Non-smokers	Reference	-
	Smokers	2.79 (-6.97; 12.6)	0.6
-	Ex-smokers	0.97 (-14.4; 16.3)	0.9
**Physical** **activity**	No or occasionally	Reference	-
	1 to 2 times per week	-3.14 (-11.8; 5.46)	0.4
	3 times or more per week	-12.4 (-25.7; 0.89)	0.06
**MH** **problems**	Yes	-1.98 (-12.5; 8.50)	0.7
	No	Reference	-
**Main** **practice** **type**	University hospital	Reference	-
	Private Community hospital	0.68 (-9.62; 10.9) 0.18 (-17.4; 17.4)	0.9 0.9
**Years** **experience^#^**	0 to 5	Reference	-
	6 to 15	-8.12 (-21.1; 4.82)	0.2
	16 or more	-10 (-29.7; 9.67)	0.3
**Working** **hours** **per** **week**	≤40	Reference	-
	41 to 50	5.01 (-8.85; 18.9)	0.5
	>50	15.6 (0.88; 30.3)	**0.04**
**Night** **shifts** **per** **month**	0	Reference	-
	1 to 4	-5.96 (-20.3; 8.38)	0.4
	5 or more	4.19 (-10.3; 18.7)	0.6
**Days** **vacation** **per** **year**	≤14	Reference	-
	15 to 21	-2.89 (-15.5; 9.66)	0.6
	22 to 30	-10.6 (-22.9; 1.59)	0.08
**2^nd^ regression. Work-related burnout**	-	**Adjusted ß (95% CI)**	***P***
**Age**	-	-0.24 (-0.92; 0.44)	0.5
**Smoking** **status**	Non-smokers	Reference	-
	Smokers	1.40 (-7.92; 10.7)	0.8
**Physical** **activity**	Ex-smokers	-8.08 (-22.8; 6.61)	0.3
	No or occasionally	Reference	-
	1 to 2 times per week	-7.49 (-15.7; 0.73)	0.07
	3 times or more per week	-6.56 (-19.2; 6.12)	0.3
**MH** **problems**	Yes	4.96 (-5.05; 15)	0.3
	No	Reference	-
**Main** **practice** **type**	University hospital	Reference	-
	Private Community hospital	-4.51 (-14.4; 5.33) -2.61 (-19.3; 14)	0.4 0.8
**Years** **Experience^#^**	0 to 5	Reference	-
	6 to 15	-3.76 (-16.1; 8.6)	0.5
	16 or more	-4.08 (-22.9; 14.7)	0.7
**Working** **hours** **per** **week**	≤40	Reference	-
	41 to 50	4.95 (-8.29; 18.2)	0.5
	>50	17.7 (3.68; 31.7)	**0.01**
**Night** **shifts** **per** **month**	zero	Reference	-
	1 to 4	-4.41 (-18.1; 9.29)	0.5
	5 or more	9.45 (-4.41; 23.3)	0.2
**Days** **vacation** **per** **year**	≤14	Reference	-
	15 to 21	0.38 (-11.6; 12.4)	0.9
	22 to 30	-9.76 (-21.4; 1.93)	0.1
**3^rd^ regression. Client-related burnout**	-	**Adjusted ß (95% CI)**	***P***
**Age**	-	-0.04 (-0.35; 0.26)	0.8
**Working** **hours** **per** **week**	≤40	Reference	-
	41 to 50	-1.05 (-12.8; 10.8)	0.9
	>50	5.59 (-6.47; 17.6)	0.4
**Night** **shifts** **per** **month**	zero	Reference	-
	1 to 4	2.65 (-9.48; 14.8)	0.7
	5 or more	15.3 (3.86; 26.7)	**0.009**

#After last year anaesthesia training (including fellowship), values in bold indicate statistical significance at *P* < 0.05; CI, confidence interval; *P, P* value; CBI, Copenhagen Burnout Inventory; MH, Mental health

**Table 5. Practical Implications for Reducing Burnout Among Anaesthesiologists in Conflict-affected Settings:** 

**Level**	**Key interventions**	**Expected impact**
**Policy**	Enforce working-hour limits (e.g., 48h/week as in EU directive)^44^ Develop national wellness and retention policies	Reduced chronic fatigue Alignment with international safety standards
**Organizational**	Ensure protected rest periods consecutive and consecutive night shifts, adapted to staffing shortages in resource-limited settings. Use flexible staffing models to address physician emigration, including workload redistribution and surge capacity planning. Improve fair recognition and compensation^45^ to support retention amid economic and workforce migration pressures Provide leadership training^9,46^ focused on crisis management and supporting staff under prolonged system strain	Lower workload-driven stress Improved morale, equity, and resilience
**Individual**	Promote autonomy, teamwork, and open communication Encourage early detection and help-seeking for burnout ^45^ Provide accessible mental health support Encourage healthy lifestyle measures (physical activity, sleep hygiene, Mediterranean diet)^41^	Enhanced job satisfaction Prevention of burnout progression
**Disaster** **preparedness**	Conduct regular disaster-preparedness training tailored to conflict-affected and resource-limited settings. Implement simulation-based crisis management exercises reflecting mass casualty and prolonged crisis scenarios. Establish structured institutional support systems for healthcare workers during mass casualty or conflict events and sustained conflict conditions, including surge capacity and psychological support	Greater resilience in crisis Improved patient safety as well as quality of care
